# Invasion dynamics of the disease vector *Aedes japonicus* in Spain

**DOI:** 10.1038/s41598-026-49121-x

**Published:** 2026-05-06

**Authors:** Federica Lucati, Fatima Chaoui, Maria Miranda Gómez, Jenny Caner, Katja Adam, Nikoleta Anicic, Karin Bakran-Lebl, Jesús F. Barandika, Manuel Barrón, Luisa Barzon, Norbert Becker, Aitor Cevidanes, Isra Deblauwe, Sarah Delacour-Estrella, Eleonora Flacio, Federica Gobbo, Mikel Alexander González, Adolfo Ibáñez-Justicia, Mihaela Kavran, Ana Klobučar, Marion Koopmans, Kornélia Kurucz, Paul T. Leisnham, Motoyoshi Mogi, Fabrizio Montarsi, Ignacio Ruiz-Arrondo, Francis Schaffner, Anna Schneider, Zoltán Soltész, Nobuko Tuno, Wim Van Bortel, Katie M. Westby, Roger Eritja, John R.B. Palmer, Frederic Bartumeus, Marc Ventura

**Affiliations:** 1 Department of Political and Social Sciences, Universitat Pompeu Fabra (UPF), Barcelona, Spain; 2 Centre for Advanced Studies of Blanes (CEAB-CSIC), Blanes, Spain; 3 Faculty of Sciences, Universitat de Girona, Girona, Spain; 4 Department of Biodiversity, Faculty of Mathematics, Natural Sciences and Information Technologies, University of Primorska, Koper, Slovenia; 5 Institute of Microbiology, University of Applied Sciences and Arts of Southern Switzerland (SUPSI), Mendrisio, Switzerland; 6 Department for Vector-Borne Diseases, AGES - Austrian Agency for Health and Food Safety, Vienna, Austria; 7 Animal Health Department, NEIKER-Basque Institute for Agricultural Research and Development, Basque Research and Technology Alliance (BRTA), Derio, Bizkaia Spain; 8 Laboratorio Agroalimentario, Gobierno de Navarra, Villava, Spain; 9 Department of Molecular Medicine, University of Padova, Padova, Italy; 10 Center for Organismal Studies, University of Heidelberg, Heidelberg, Germany; 11 Unit of Entomology, Department of Biomedical Sciences, Institute of Tropical Medicine, Antwerp, Belgium; 12 Department of Animal Pathology, Faculty of Veterinary Sciences, Instituto Agroalimentario de Aragón-IA2 (Universidad de Zaragoza-CITA), Zaragoza, Spain; 13 Laboratory of Medical Entomology and Vector-borne Diseases, Istituto Zooprofilattico Sperimentale delle Venezie, Legnaro, Italy; 14 Departamento de Soluciones Ambientales y Entomología, Grupo SASTI, Sevilla, Spain; 15 CIBER de Epidemiología y Salud Pública (CIBERESP), Madrid, Spain; 16 Centre for Monitoring of Vectors, Netherlands Institute for Vectors, Invasive plants and Plant Health (NIVIP), Netherlands Food and Consumer Product Safety Authority (NVWA), Wageningen, The Netherlands; 17 Centre of Excellence-One Health, Vectors and Climate, Faculty of Agriculture, University of Novi Sad, Novi Sad, Serbia; 18 Department of Epidemiology, Andrija Štampar Teaching Institute of Public Health, Zagreb, Croatia; 19 Viroscience Department and Pandemic and Disaster Preparedness Research Centre, Erasmus MC, Rotterdam, The Netherlands; 20 National Laboratory of Virology, Szentágothai Research Centre, University of Pécs, Pécs, Hungary; 21 Institute of Biology, Faculty of Sciences, University of Pécs, Pécs, Hungary; 22 Department of Environmental Science and Technology, University of Maryland, College Park, MD USA; 23 Division of Parasitology, Faculty of Medicine, Saga University, Saga, Japan; 24 Centre of Rickettsiosis and Arthropod-Borne Diseases, Hospital Universitario San Pedro-CIBIR, Logroño, Spain; 25 BioSys - EI Schaffner Francis, Steinbach, France; 26 Institute of Ecology and Botany, HUN-REN Centre for Ecological Research, Vácrátót, Hungary; 27 National Laboratory for Health Security, HUN-REN Centre for Ecological Research, Budapest, Hungary; 28 School of Natural Science and Technology, Kanazawa University, Kanazawa, Japan; 29 Tyson Research Center, Washington University in Saint Louis, Eureka, MO USA; 30 Catalan Institution for Research and Advanced Studies (ICREA), Barcelona, Spain; 31 Centre for Ecological Research and Forestry Applications (CREAF), Cerdanyola del Vallès, Spain

**Keywords:** *Aedes*, Invasive mosquitoes, Invasion genetics, Northern Spain, Points of entry, *wsp* marker, Ecology, Ecology, Zoology

## Abstract

Native to East Asia, the Asian bush mosquito (*Aedes japonicus*) has recently expanded its global range, with established invasive populations in Europe and North America. Given its potential role as a vector of various arboviruses, understanding its invasion process and ecological dynamics is crucial for managing its spread and mitigating public health risks. In the Iberian Peninsula, the species was first detected in Asturias in 2018 and has since expanded to neighbouring regions. Here, we elucidate the invasion pathways and possible origins of *Ae. japonicus* populations in Spain using sequence data and microsatellite markers, and by screening for the presence of maternally transmitted bacteria of the genus *Wolbachia*. We analysed 635 *Ae. japonicus* from 14 countries, including Japan (native range), the United States, and 12 European countries. No clear association between haplotypes and geographical location was detected in any of the three genes analysed (nuclear ITS2, mitochondrial COI and ND4). *Wolbachia* was not detected in any of the screened samples. In contrast, microsatellite-based population structure analyses showed that most Spanish samples clustered closely with those from College Park, Maryland (USA), located near the Port of Baltimore, one of the largest ports in the United States and a recognised gateway for invasive species introductions. Northern Spain hosts major seaports such as Bilbao and Gijón, whereas the nearest established *Ae. japonicus* population in Europe lies over 1,000 km away in northeastern France. Taken together, these findings suggest that the most plausible invasion route of *Ae. japonicus* into Spain involves maritime transport from the eastern coast of the United States to northern Spanish ports, likely accompanied by additional minor introductions of European origin. The inclusion of additional microsatellite loci originally developed for *Ae. albopictus* yielded results consistent with those obtained using *Ae. japonicus*-specific loci, reinforcing the robustness of the observed patterns. This work provides new insights into the invasion process of *Ae. japonicus* in Europe and highlights the need for continuous monitoring and tailored interventions at key ports of entry.

**Supplementary Information:**

The online version contains supplementary material available at 10.1038/s41598-026-49121-x.

## Introduction

Biological invasions have a significant impact at the ecological, economic, and social levels^1,2^. In the case of mosquitoes, invasions can pose serious public health risks due to their role as disease vectors^3,4^. Invasive mosquito species of the genus *Aedes* have spread globally, facilitated by human activities like international trade and travel^5,6^. These mosquitoes are highly adaptable, capable of thriving in diverse habitats—e.g. from tropical rainforests to urban environments, as in the case of the Asian tiger mosquito *Aedes albopictus*—by exploiting artificial breeding sites like discarded tyres and containers with standing water. Their introduction into new regions can lead to the emergence of new disease transmission cycles, presenting challenges for public health^3^.

The Asian bush mosquito (*Aedes japonicus*) is native to temperate East Asia, primarily Japan and Korea^7^. In recent years, human activity—particularly the international trade of used tyres—has facilitated its spread to other parts of the world, mainly North America and Europe^8^. It first reached Europe in 2000, when it was detected in Normandy, France^9^. Two years later, it was found in Belgium, likely introduced via the used tyre trade^10^. Since then, *Ae. japonicus* has expanded its range across several European countries, including Austria, Croatia, France, Germany, Hungary, Italy, Luxembourg, the Netherlands, Poland, Russia, Slovenia, Serbia, Spain, and Switzerland^11–13^. Unlike *Ae. albopictus* and the yellow fever mosquito (*Aedes aegypti*), *Ae. japonicus* is better adapted to colder and more temperate climates, which has supported its spread across central Europe^14^. Furthermore, this species is less urban in its habitat preferences, and it is commonly found in rural areas dominated by deciduous forests. It breeds in a wide variety of natural and artificial water-holding containers, such as catch basins, cattle troughs, ground puddles, rock pools, buckets, tree holes, and tyres^4,11,15^. While *Ae. japonicus* is considered a lesser public health threat than *Ae. albopictus* or *Ae. aegypti*, it remains of epidemiological interest. In the field in the United States, it is mostly associated with the transmission of West Nile virus^16^, La Crosse virus^17^ and Cache Valley virus^18^. Laboratory studies have demonstrated its vector competence for several other arboviruses, including Japanese encephalitis, Rift Valley fever and West Nile viruses, and, to a lesser extent, dengue, chikungunya, Zika and Usutu viruses^19–24^.

*Aedes japonicus* has become the third invasive mosquito species with vector competence recorded in Europe, following *Ae. albopictus* and *Ae. aegypti*, and the second of these to become established in Spain. The citizen science platform Mosquito Alert enabled the first detection of *Ae. japonicus* in Spain during the summer of 2018, in the region of Asturias^25^, and two years later, the species had already been detected in two neighbouring regions (Cantabria and the Basque Country)^15^. Currently, *Ae. japonicus* is found along much of the northern coast of Spain, with established populations in Asturias, Cantabria, the Basque Country and Navarre^26–28^, creating a new colonization area in Europe. Interestingly, the *Ae. japonicus* cluster detected in northern Spain is geographically isolated from the main invasion hotspot in central Europe, raising questions about its actual invasion pathway. At the time of discovery, the nearest known European population was located approximately 1,000 km away, in northeastern France.

Here, our aim was to elucidate the invasion pathway and the potential origin of the *Ae. japonicus* populations recently established in northern Spain. To address this question, we conducted a large-scale population genetics study of the species, integrating information from mitochondrial (mtDNA) and nuclear (nDNA) markers, along with seven microsatellite loci. To explore whether resolution could be improved, a subset of samples was also genotyped at 13 additional loci developed for *Ae. albopictus*. We also considered the possible influence of *Wolbachia*, endosymbiotic bacteria known to affect mtDNA diversity in many arthropods, including *Ae. albopictus*^29–31^, to ensure a reliable interpretation of mitochondrial patterns. Altogether, this framework allowed us to reconstruct the introduction history of *Ae. japonicus* in Spain and to situate these populations within the broader European invasion scenario.

## Methods

### Sample collection, DNA extraction and marker choice

**Fig. 1:**
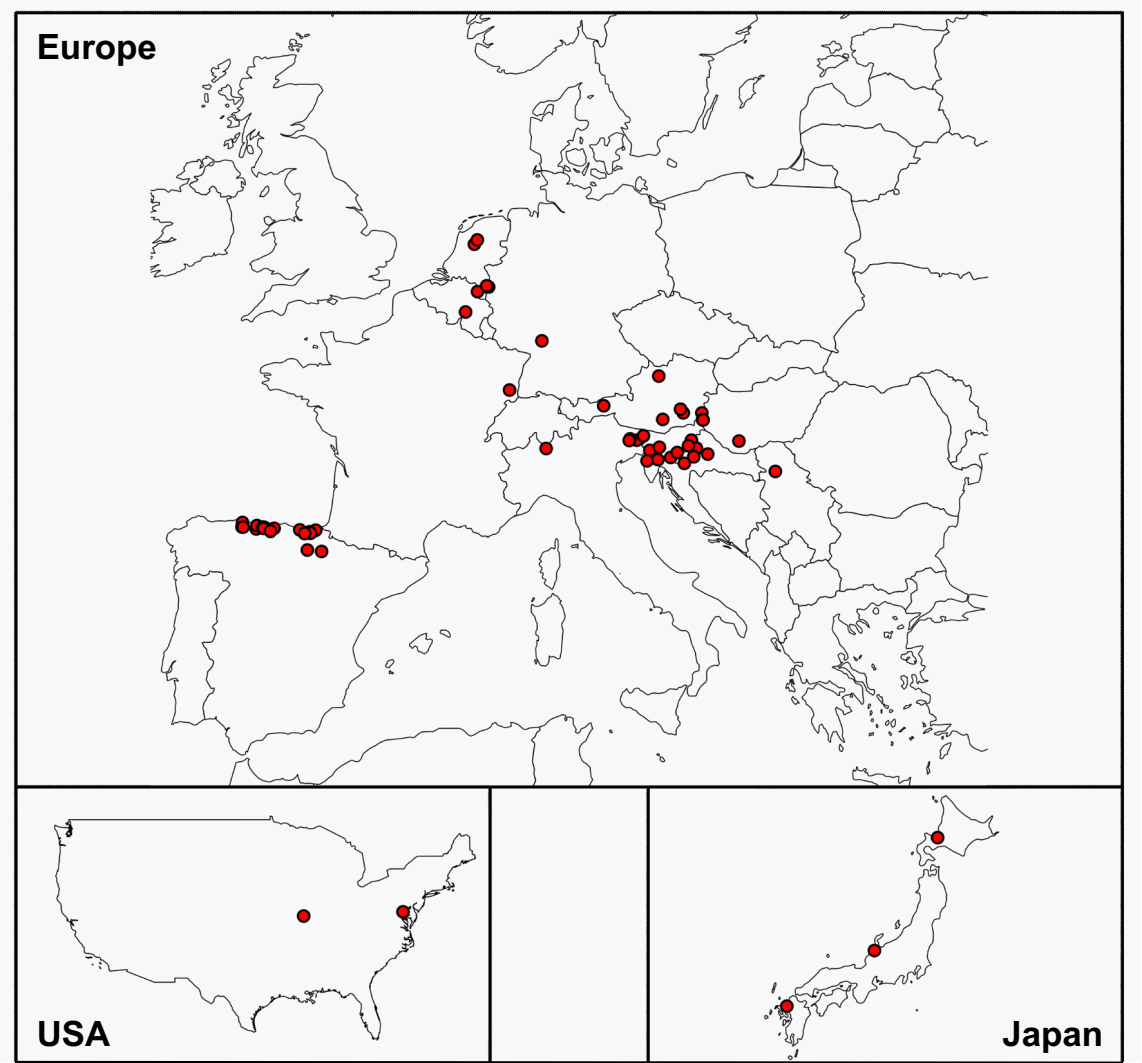
Geographic location of sampling sites for *Aedes japonicus*. A total of 62 sites (shown as red dots) were sampled across Europe, the United States, and Japan. For further information see Supplementary Table S1.

A total of 635 *Ae. japonicus* specimens were collected between 2018 and 2023 at 62 locations, encompassing 14 countries: Japan, where the species is native, the United States of America, the first country in which the species is known to have become established outside its native range, and 12 European countries including Spain (Fig. 1; Table 1 and Supplementary Table S1). The Spanish samples were obtained from the autonomous communities of Navarre, the Basque Country, Cantabria and Asturias, spanning the entire known distribution range of *Ae. japonicus* in the country. Specimens were collected as adults, larvae, or adults reared from eggs or larvae (Supplementary Table S1), and stored in either absolute or 70% ethanol at −20 °C until DNA extraction.

Genomic DNA was extracted from the whole bodies of mosquitoes using the DNeasy Blood and Tissue kit (Qiagen, Hilden, Germany) following the manufacturer’s protocol, in a final volume of 100 µL.

Microsatellites were prioritised as primary genetic markers because of their higher level of polymorphism and resolution for fine‑scale population and invasion analyses. Given budget constraints, gene regions were included only for a subset of samples to complement microsatellite data, and all available GenBank sequences were incorporated to increase marker coverage (Table 1 and Supplementary Table S1). The uneven sequencing effort for gene regions reflects the prioritisation of Spanish samples and countries under‑represented in GenBank.

**Table 1:** Number of samples analysed per country and genetic marker: ITS2 (N_ITS2_), COI (N_COI_), ND4 (N_ND4_), *Wolbachia* presence (N_*Wolbachia*_) and microsatellites (N_µsat_). Genetic diversity parameters inferred from microsatellites: allelic richness (A_R_), private allelic richness (PA_R_), observed (Ho) and expected (He) heterozygosity, and inbreeding coefficient (F_IS_). See Supplementary Table S1 for details on sampled localities. - indicates no samples available.

Country	N_ITS2_	N_COI_	N_ND4_	N_*Wolbachia*_	N_µsat_	A_R_	PA_R_	Ho	He	F_IS_
Spain	5	24	37	41	166	3.00	0.07	0.40	0.43	0.07
France	1	4	6	6	20	3.08	0.02	0.53	0.49	−0.04
Italy	9	10	9	12	48	3.06	0.04	0.45	0.43	−0.04
Switzerland	-	-	-	-	19	3.23	0.12	0.34	0.48	0.32
Belgium	-	-	-	-	27	3.51	0.14	0.37	0.48	0.24
The Netherlands	1	5	5	6	47	3.97	0.16	0.46	0.57	0.22
Germany	-	-	-	-	14	3.70	0.08	0.50	0.53	0.09
Austria	2	5	5	5	58	4.03	0.27	0.43	0.54	0.22
Hungary	4	8	10	12	51	3.79	0.07	0.44	0.50	0.13
Slovenia	-	-	-	-	37	3.40	0.06	0.46	0.46	0.03
Croatia	-	-	-	-	36	3.42	0.11	0.53	0.51	−0.03
Serbia	-	-	-	-	19	3.28	0.05	0.49	0.54	0.13
Japan	5	9	15	18	66	3.83	0.14	0.42	0.51	0.17
USA	4	5	9	12	27	3.32	0.14	0.41	0.46	0.13
N total and average of parameters	31	70	96	112	635	3.47	0.11	0.45	0.50	0.12

### Nuclear and mitochondrial gene sequencing and analysis

A subset of samples belonging to eight countries was analysed for three gene regions (Table 1 and Supplementary Table S1), including one nuclear ribosomal gene (second internal transcribed spacer of ribosomal DNA – ITS2) and two mitochondrial fragments (cytochrome *c* oxidase gene subunit 1 – COI, and NADH dehydrogenase subunit 4 gene and adjacent tRNAs – ND4). ND4 and COI have been extensively used in *Aedes* population genetics and invasion studies due to their high variability^32–35^, while ITS2 was included to incorporate nuclear variation and avoid relying solely on maternally inherited mtDNA, which can be influenced by factors such as *Wolbachia*^29^. Primers used for amplification and sequencing were: for ITS2, ITS-CP-P1A (5′-GTGGATCCTGTGAACTGCAGGACACATG-3′) and ITS-CP-P1B (5′-GTGTCGACATGCTTAAATTTAGGGGGTA-3′)^36^, for COI, LCOI490 (5′-GGTCAACAAATC ATAAAGATATTGG-3′) and HCO2198 (5′-TAAACTTCAGGGTGACCAAAAAATCA-3′)^37^, and for ND4, N4J-8502D (5′-CGTAGGAGGAGCAGCTATATT-3′) and N4N-8944D (5′-AAGGCTCATGTTGAAGCTCC-3′)^33^. Amplification conditions for ITS2 consisted of 95 °C for 3 min followed by 35 cycles of 95 °C for 30 s, 37 °C for 30 s and 72 °C for 1 min, and a final extension of 72 °C for 15 min. For COI, PCR cycling conditions were: a denaturing step of 95 °C for 3 min, 5 cycles of 95 °C for 30 s, 45 °C for 30 s and 72 °C for 1 min, followed by 30 cycles of 95 °C for 30 s, 50 °C for 30 s and 72 °C for 1 min, and a final extension of 72 °C for 15 min. As for ND4, we applied a denaturing step of 95 °C for 3 min followed by 35 cycles of 95 °C for 30 s, 55 °C for 30 s and 72 °C for 1 min, and a final extension of 72 °C for 15 min. PCR products were purified using a NucleoFast 96 PCR Plate (Macherey-Nagel) and sequenced bidirectionally on an ABI 3730 capillary sequencer (Secugen, Madrid, Spain).

Resulting sequences were aligned in MEGA 11^38^ using the ClustalW algorithm with default settings. For every analysed gene region, genealogic relationships among haplotypes were estimated using Haploviewer^39^. The optimal nucleotide-substitution model was defined by jModelTest 2.1.3^40^ under the Akaike Information Criterion (AIC). Phylogeny among haplotypes was then estimated with RAxML 7.7.1^41^ and the best generated tree was selected for haplotype network construction in Haploviewer, based on sequences retrieved from GenBank and this study. RAxML was run with a GTRCAT model of rate heterogeneity for ITS2, and a GTR + I model for ND4 and COI, applying 1,000 bootstrap replicates. Genetic diversity parameters, i.e. number of haplotypes (H) and polymorphic sites (S), as well as haplotype (Hd) and nucleotide (Π) diversity indices, were calculated for every gene region in DNASP 6.12.03^42^.

### Wolbachia detection

To examine whether mtDNA diversity could be affected by the presence of endosymbiotic bacteria of the genus *Wolbachia*, 112 randomly selected samples from eight countries were analysed for the *wsp* gene (Table 1). Primers 81 F (5′-TGGTCCAATAAGTGATGAAGA-3′) and 691R (5′-AAAAATTAAACGCTACTCCA-3′) were used for amplification and sequencing^43^. Amplification conditions consisted of a denaturing step of 95 °C for 3 min, followed by 30 cycles of 95 °C for 1 min, 55 °C for 1 min and 72 °C for 1 min, and a final extension at 72 °C for 3 min^44^. For each sample, two PCR replicates were run in order to validate the results ^45^. A third replicate was run only for samples that showed incongruent results based on the two prior replicates. *Wolbachia* infection was confirmed by two successful amplifications of the *wsp* marker. One positive and one negative control were included on each plate. The positive controls consisted of *Ae. albopictus* DNA extracts that had produced a positive result in a previous study^46^. The negative controls consisted of reactions containing no DNA template.

### Microsatellite screening and analysis

All samples were screened for a set of seven microsatellite loci specific for *Ae. japonicus* distributed in one multiplex^47^ (Table 1, Supplementary Tables S1 and S2). PCR amplification conditions followed^47^. In addition, to explore whether resolution could be improved, a subset of samples was also genotyped at 13 loci originally developed for *Ae. albopictus*, for a total of 20 loci spread in 3 multiplexes (Supplementary Table S2). The first multiplex included the seven *Ae. japonicus* loci from^47^, while the second and third multiplexes contained 13 *Ae. albopictus* loci from^48,49^. *Ae. albopictus* loci were selected for their clear cross-amplification in *Ae. japonicus* and high variability. The program Multiplex Manager 1.2^50^ was employed to plan and optimize the distribution of the second and third multiplexes. PCR amplification conditions were the same for all multiplexes: 95 °C for 15 min followed by 35 cycles of 94 °C for 30 s, 58 °C for 1 min 30 s and 72 °C for 1 min, and a final extension of 72 °C for 30 min. Amplified fragments were sized with LIZ-500 size standard and binned using Geneious Prime 2024.0.5^51^. Loci that presented > 20% of missing data, as well as samples that could not be reliably scored for at least 80% of the loci, were excluded from further analyses.

Using GENEPOP 4.2^52^, we tested for linkage disequilibrium among loci to identify non-random associations of alleles, and for deviations from Hardy–Weinberg equilibrium (HWE) across loci and populations to assess potential effects of evolutionary forces such as genetic drift, inbreeding or selection. The Bonferroni correction was applied to adjust for multiple comparisons (α = 0.05^53^. Genetic diversity parameters, i.e. expected (He) and observed (Ho) heterozygosity and inbreeding coefficient (Fis), were calculated at the country level using Genetix 4.05^54^. To further investigate genetic diversity and distinctiveness, HP-Rare 1.1^55^ was used to estimate allelic richness (Ar) and private allelic richness (PAr).

The likely origin of Spanish *Ae. japonicus* and overall population structure were investigated using three different approaches: (1) a Principal Component Analysis (PCA) using the ADEGENET package 2.1.1^56^ in R 4.4.0; (2) a Bayesian cluster analysis implemented in STRUCTURE 2.3.4^57^; and (3) a neighbour-joining (NJ) tree using the program POPTREE2^58^. As for STRUCTURE analysis, we conducted 20 simulations for each K, set from 1 to the number of populations plus one, with 100 K burn-in steps followed by 500 K Markov chain Monte Carlo repetitions. The program was run using the admixture model with correlated allele frequencies. StructureSelector^59^ was used to determine the optimal number of genetic clusters according to the ΔK method^60^, as well as to average replicate runs of the optimal K and plot the final output. As for the NJ tree, we used Nei’s genetic distance (D_A_^61^ and performed 1,000 bootstraps. In the NJ tree, samples were grouped at the regional level to account for within-country variability (Supplementary Table S1).

## Results

### Nuclear and mitochondrial genes

**Fig. 2:**
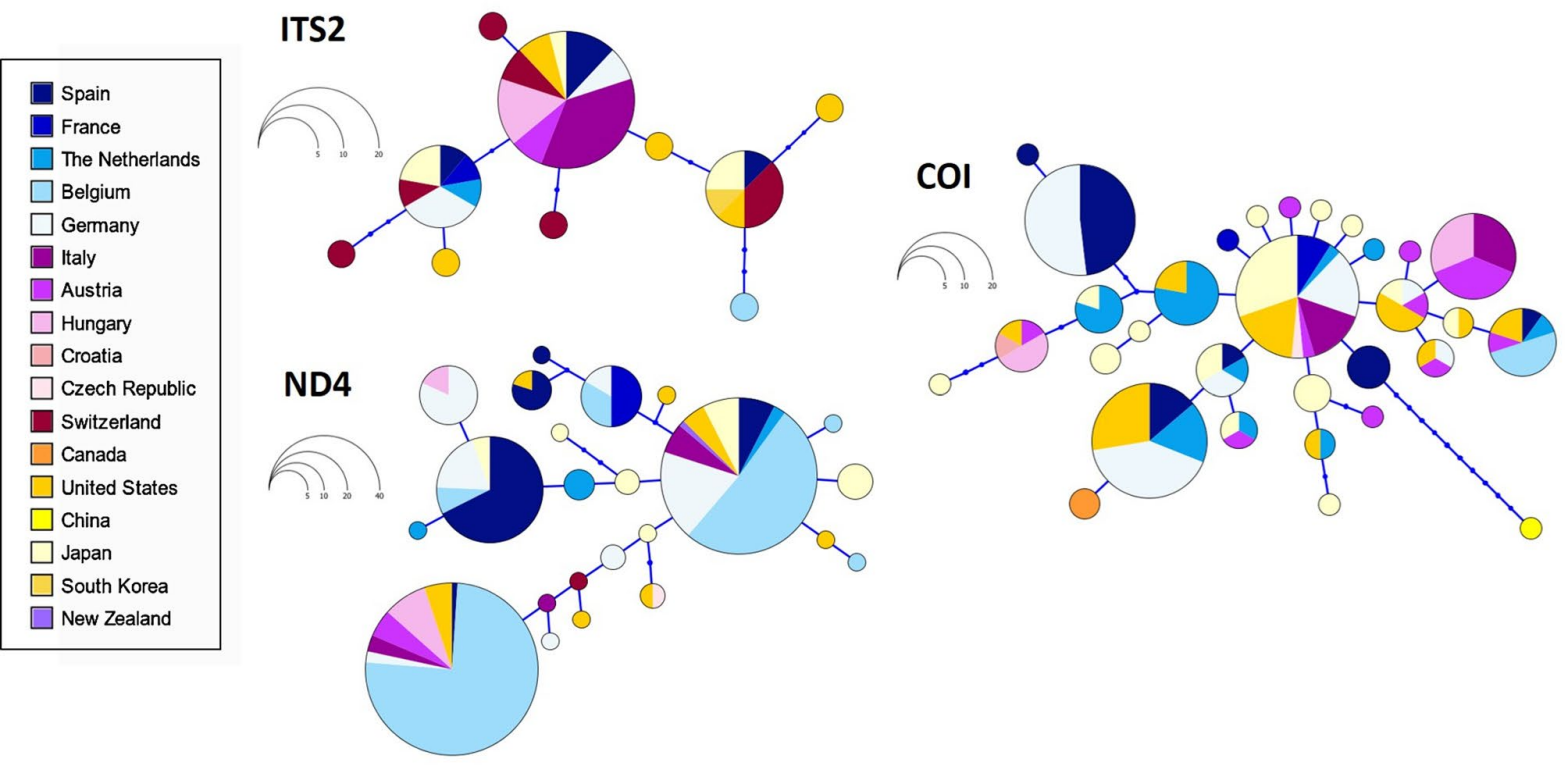
Haplotype networks for each analysed genetic marker (ITS2, ND4, and COI), including newly generated sequences and sequences retrieved from GenBank. Each circle represents a unique haplotype and the circle area is proportional to the number of sequences of a given haplotype. No clear geographic association was detected between Spanish samples (shown in dark blue) and those from other countries for any of the three markers analysed.

The newly assembled (excluding sequences retrieved from GenBank) nuclear DNA alignment (ITS2) comprised 31 sequences of 336 bp, whereas the mitochondrial DNA alignments included 70 and 96 sequences of 635 and 418 bp for COI and ND4, respectively (Table 1). Overall, no clear association was found between haplotypes and geographic distribution in any of the analysed genes. All haplotype networks revealed the presence of a few dominant haplotypes and a lack of evident genetic structure across the studied countries, with no apparent association with the Spanish samples either (Fig. 2). Overall haplotype and nucleotide diversities were 0.49 ± 0.08 and 0.0031 ± 0.00065 for ITS2, respectively, 0.90 ± 0.01 and 0.0052 ± 0.00027 for COI, and 0.76 ± 0.02 and 0.0056 ± 0.00020 for ND4. We found 10 haplotypes defined by 10 polymorphic sites (2 parsimony informative) for ITS2, 32 haplotypes defined by 32 polymorphic sites (15 parsimony informative) for COI, and 23 haplotypes defined by 20 polymorphic sites (17 parsimony informative) for ND4.

### *Wolbachia* screening

All analysed samples (112) tested negative for *Wolbachia* based on amplification of the *wsp* marker.

### Microsatellites

After filtering for missing data, all seven loci specific for *Ae. japonicus* and a total of 635 samples were retained for analyses. A subset of samples (375) belonging to eight countries was also genotyped at 13 additional loci developed for use on *Ae. albopictus*; two of these loci were discarded due to poor amplification (Supplementary Table S2). No significant linkage disequilibrium or departures from Hardy–Weinberg equilibrium across sampled localities and loci were observed after applying the Bonferroni correction. Genetic diversity parameters calculated at the country level are shown in Table 1.

**Fig. 3:**
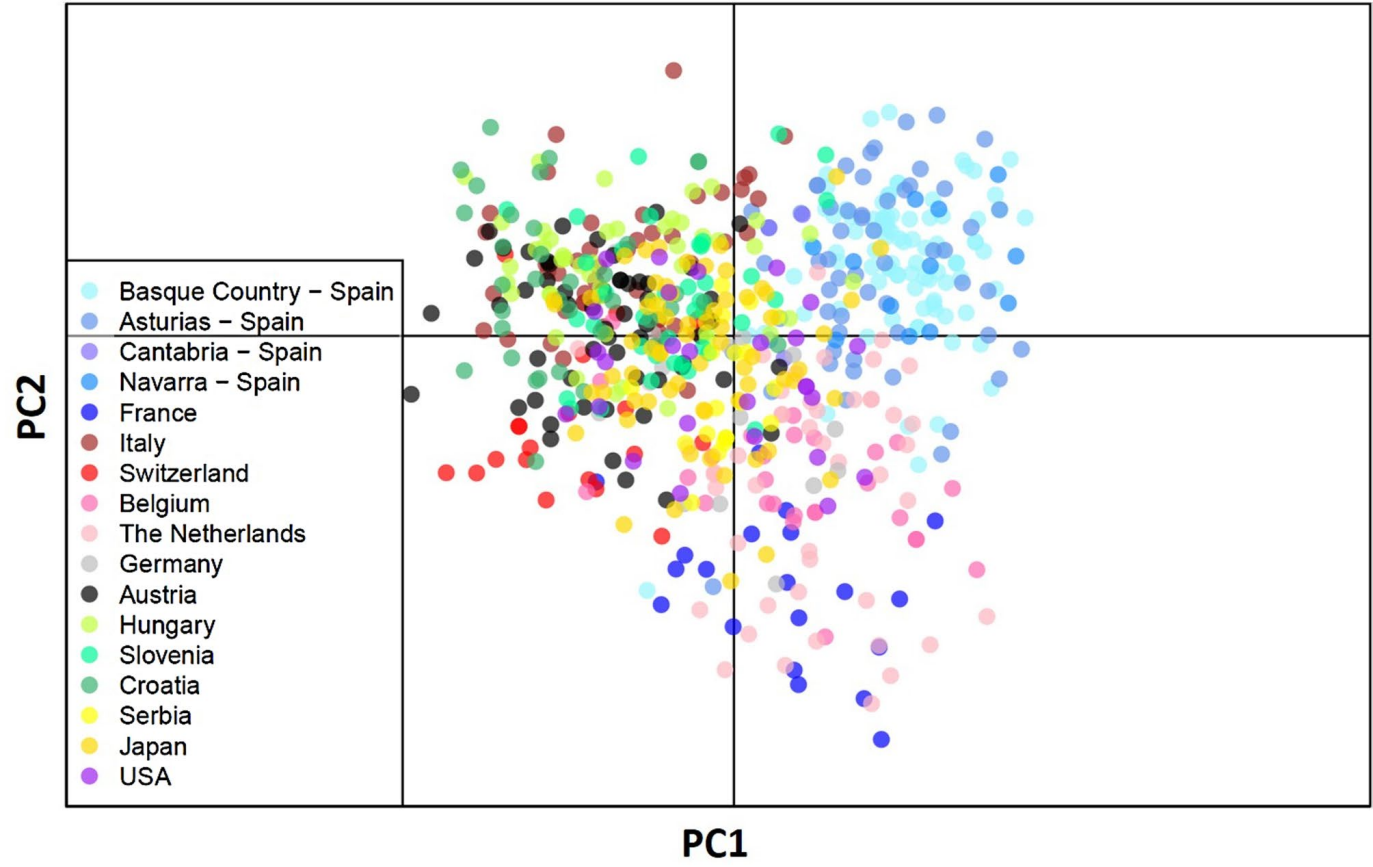
Principal Component Analysis (PCA) of *Aedes japonicus* samples based on microsatellite data. Each point represents a single individual. Samples are coloured according to their geographic origin—by region for Spanish populations and by country for all other samples.

Population structure analysis based on the seven *Ae. japonicus* microsatellites revealed a clearer picture of geographic association compared to gene sequence analyses. In the PCA, most of the Spanish samples clustered closely together in the upper right corner (represented by the light-blue shades in Fig. 3), exhibiting a distinct genetic identity, with little overlap or mixing with samples from other countries. A few individuals, mostly from the Basque Country and Asturias regions, overlapped with samples from other countries at the edges of the Spanish cluster. In addition, two samples appeared in the lower central area of the PCA plot, completely separated from the main Spanish group. Among the other European countries, a certain separation was observed between Italy, Austria, Hungary, Slovenia and Croatia (upper left corner), and France, Belgium and the Netherlands (lower area of the PCA).

**Fig. 4:**
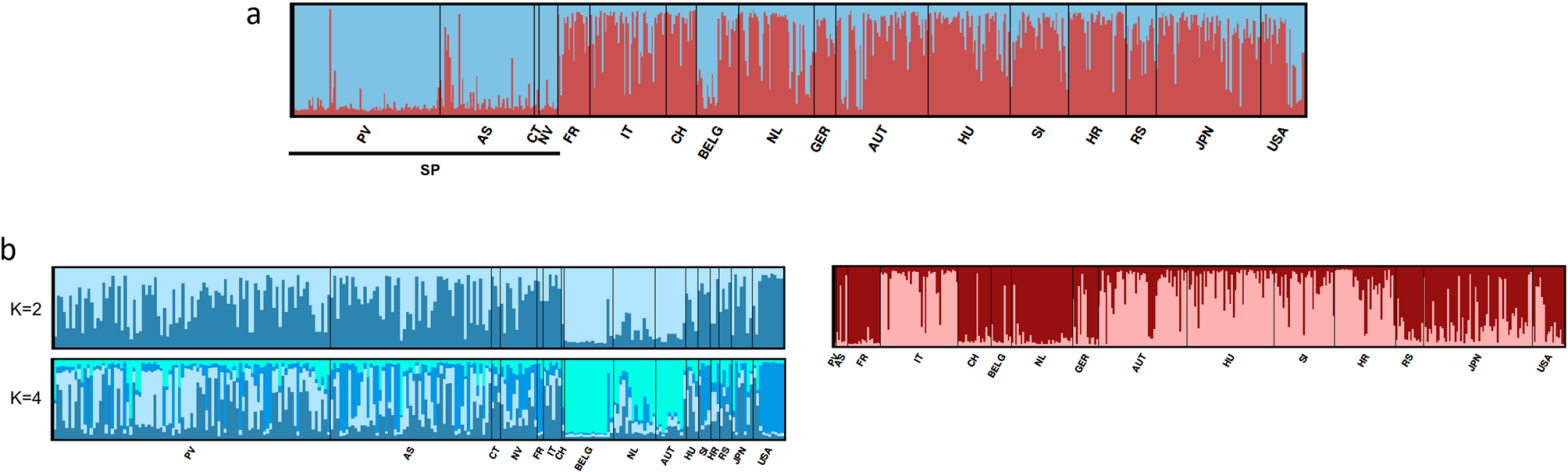
Results of Bayesian clustering analysis of *Aedes japonicus* based on microsatellite data. (**a**) STRUCTURE barplot showing individual membership probabilities for K = 2 across all sampled individuals. Each individual is represented by a vertical bar corresponding to the sum of assignment probabilities to the K cluster. Black lines separate countries, or regions in the case of Spain. (**b**) Substructure detected within the two main genetic clusters of Fig. 4a (blue cluster at K = 2 and K = 4, red cluster at K = 2). Country/Spanish region codes: SP: Spain; FR: France; IT: Italy; CH: Switzerland; BELG: Belgium; NL: The Netherlands; GER: Germany; AUT: Austria; HU: Hungary; SI: Slovenia; HR: Croatia; RS: Serbia; JPN: Japan; USA: United States; PV: Basque Country; AS: Asturias; CT: Cantabria; NV: Navarra.

STRUCTURE analysis identified K = 2 as the best clustering solution (Fig. 4a and Supplementary Fig. S1). The first cluster grouped nearly all Spanish samples together with one population from the United States (College Park - Maryland), one population from Belgium and a few scattered samples from other European countries, mostly the Netherlands and Austria (light blue cluster in Fig. 4a). All remaining samples were assigned to the second cluster (red in Fig. 4a). When each cluster was analysed separately, additional substructure emerged (Fig. 4b and Supplementary Fig. S1): the first (light blue) cluster showed a major peak at K = 4 and a secondary peak at K = 2, while the second (red) cluster divided into two sub-clusters. Within the first light blue cluster, samples from Belgium, Austria and most of the Netherlands displayed a different genetic ancestry compared with Spanish and US samples. Within the second red cluster, two groups were evident: a central-eastern European group (Italy, Austria, Hungary, Slovenia and Croatia) and a central-western European group (France, Switzerland, Belgium, the Netherlands and Serbia), with Germany containing individuals of both lineages. Of the seven Spanish samples belonging to the red cluster (all from the Basque Country and Asturias), two from Asturias clustered with the central-eastern European group, while the remaining clustered with the central-western cluster.

**Fig. 5:**
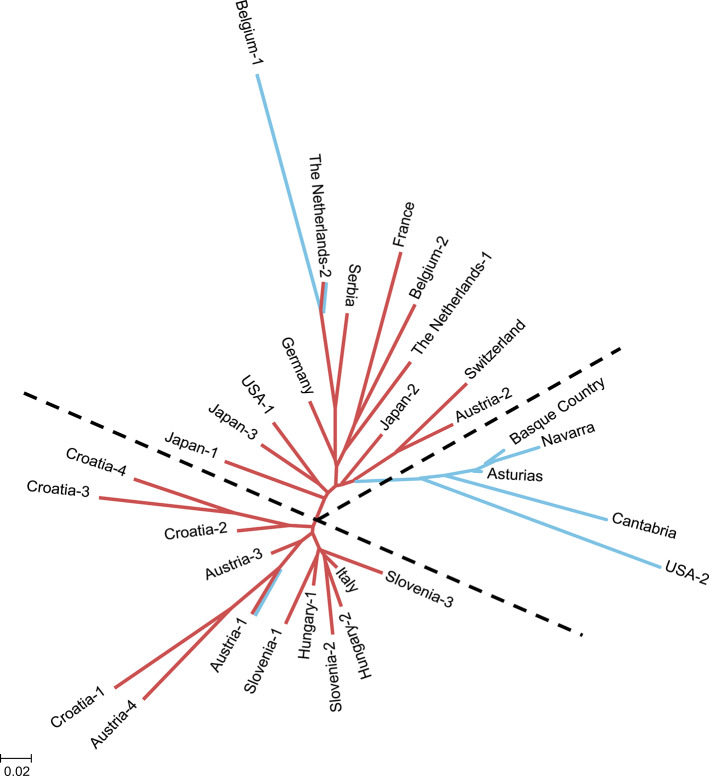
Neighbour-joining tree over all *Aedes japonicus* sampled regions based on microsatellite data. Branch colours delineate the two main genetic clusters identified by STRUCTURE analysis (see Fig. 4a). Dashed lines indicate the separation between the central-eastern and central-western European lineages found within the red cluster (see Fig. 4b). Note the clustering of Spanish samples with the US samples of College Park (USA-2; light blue branches). See Supplementary Table S1 for sample codes.

The NJ tree run at the regional level showed an even stronger affinity between the Spanish samples and the US samples of College Park (labelled USA-2 in Fig. 5). Moreover, consistent with PCA and STRUCTURE analyses, a clear separation was observed between the central-eastern and central-western European groups. Altogether, results for Spain point to a primary introduction route linking populations from Maryland with those in Spain, alongside additional, smaller introduction pathways within Europe.

Including the 13 *Ae. albopictus* loci resulted in similar but slightly higher-resolution outcomes in population structure analyses (i.e., PCA and STRUCTURE). In particular, the PCA showed a clearer separation of most of the Spanish samples from the rest (Supplementary Fig. S2), as well as a more straightforward separation at K = 2 as for STRUCTURE analysis (Supplementary Fig. S3). Nevertheless, given the limited improvement in resolution and the increased costs and processing time associated with the additional loci, we considered the use of *Ae. japonicus*-specific microsatellites to be a more efficient and cost-effective approach for large-scale analyses, as the overall conclusions remained largely unchanged.

## Discussion

Invasive *Aedes* mosquitoes are well known for their capacity to spread globally through human-mediated transport. Long-distance dispersal typically occurs via international commercial shipping, particularly at the egg stage, and through the movement of goods such as used tyres and ornamental plant cuttings^62^. In contrast, local or regional expansion, especially for species like *Ae. albopictus*, is often facilitated by ground transportation, with adults or immature stages hitchhiking in vehicles^5,63^. The economic and public health costs associated with biological invasions are substantial, underscoring the need for effective surveillance, control, and assessment of arbovirus transmission risk^1^.

In the present study, we provide new insights into the possible origin of the *Ae. japonicus* invasion of Spain, which represents one of the most recent recognised introductions of this species in Europe^15^. Microsatellite-based analyses suggest that the species may have arrived mostly from the United States, as evidenced by the close genetic affinity between Spanish samples and those from College Park (Maryland), which is especially evident in the neighbour-joining tree. Interestingly, College Park is situated about 50 km from the commercial port of Baltimore, one of the largest ports in the United States and a potential source area for invasive species. Similarly, the northern coast of Spain—where the Iberian *Ae. japonicus* cluster is situated—also hosts major commercial ports, including those of Bilbao (Basque Country) and Gijón (Asturias).

In the United States, *Ae. japonicus* was first detected in 1998 in New York, New Jersey and Connecticut^64,65^. By 2011, the species had spread to 33 US states, including Hawaii. Early genetic analyses revealed marked differentiation between populations from New York, New Jersey and Connecticut and those detected in Maryland and Pennsylvania, pointing to two distinct foci of expansion that may have resulted from independent introductions or post-introduction divergence^33^. In Spain, *Ae. japonicus* was first reported in the summer of 2018 in the municipality of Siero (Asturias)^25^. At the time, an overseas introduction was hypothesised by the authors, given the proximity of Siero to the port of Gijón (~ 20 km), the second largest in northern Spain. Additional evidence supporting an overseas introduction is the fact that the Iberian cluster, the southernmost in Europe, is geographically isolated: the nearest known populations in northeastern France are located over 1,000 km away. No intermediate populations have been detected between these regions, and there is no evidence suggesting that *Ae. japonicus* adults disperse via road transport, despite active ovitrap surveillance along major highways in southern France^66^. Moreover, our study found no clear genetic affinity between Spanish and French populations, making a primary land-based introduction less likely. Taken together, these observations reinforce our microsatellite results and support the hypothesis that most of the Iberian *Ae. japonicus* cluster originated via maritime trade, potentially through the port of Baltimore. We analyzed trade data from the Observatory of Economic Complexity^67^ and found that between 2021 and 2024 at least 2196.7 metric tons of cargo was shipped from the US port of Baltimore to the Spanish ports of Gijón and Bilbao. Of this, 446.3 metric tons consisted of construction material, a shipment type that has been associated with the transport of *Aedes* mosquitoes^68^. Nevertheless, other hypotheses cannot be ruled out, including the possibility that populations of this species originated from previously unsampled locations in the USA. Additional sampling around the area of Baltimore, as well as across larger regions of the USA, could provide a clearer picture of the relationship between the Baltimore area and the Spanish population cluster. Nonetheless, a few individuals from the Basque Country and Asturias were genetically related to populations from other European countries, suggesting occasional connections with the EU area, possibly via maritime transport from EU harbours. However, this pathway does not appear to represent the primary route of introduction.

In Europe, at least eight distinct population clusters of *Ae. japonicus* have been identified, with Spain representing the eighth^25,69^. Among these, Belgium is the only country where the introduction pathway has been well documented, most likely linked to the transport of eggs through the used tyre trade^10^. For the other cases, information on the origin and dispersal drivers remains unavailable, although several independent introduction events have been suggested^11,12,35^. Two major introduction routes have been proposed in Europe: one covering southern Germany, Austria, Belgium, and Slovenia, and another comprising western and northern Germany^11^. In addition to these two major genotypes, less geographically widespread genotypes have been detected in some countries, e.g. the Netherlands^70^, Croatia^12^, and Belgium^35^, thus revealing distinct introduction or admixture events within the same country. Our microsatellite-based analyses instead revealed two geographically separated lineages within Europe, one covering central-eastern Europe and another in central-western Europe. Consistent with previous studies, we also detected multiple lineages within the same country, with at least two distinct genetic groups co-occurring e.g. in Belgium, Austria, and the Netherlands. In contrast, the Spanish population shows only limited genetic heterogeneity: most specimens analysed here form a compact and homogeneous group, as demonstrated by PCA, Structure and NJ analyses, and are clearly distinct from all other European populations examined. This pattern suggests a primary introduction event, likely from a single source, accompanied by additional minor invasions of probable European origin. The Spanish case may therefore represent a previously undocumented introduction pathway of *Ae. japonicus* into Europe —possibly via maritime transport from the USA— adding to the genetic makeup of the species in Europe. Determining the timing of arrival and subsequent establishment, however, remains challenging. Following its first detection in Asturias in 2018, the species was rapidly confirmed across most of northern Spain, suggesting that it had likely been present for a considerable period prior to its discovery. This reflects the well-known surveillance paradox: a recent detection does not necessarily imply a recent introduction.

In large-scale population genetic studies, it is essential to balance cost-efficiency with analytical robustness, particularly when working with several hundred samples. Microsatellite analyses are known to be sensitive to the number of loci employed, as marker number may influence the resolution of the inferred genetic structure^71^. To assess whether increasing the number of markers would enhance resolution in *Ae. japonicus*, approximately half of the samples were genotyped with 20 loci, combining seven species-specific loci and 13 loci originally developed for *Ae. albopictus*. Comparative analyses showed that the expanded dataset produced patterns largely congruent with those obtained using only the seven *Ae. japonicus* loci, although with a slightly higher resolution. This indicates that the limited set of species-specific loci is sufficient to capture the main genetic signals, providing reliable, accurate, and cost-effective resolution for population genetic studies. While the additional markers offered only marginal improvements, this highlights both the robustness of the *Ae. japonicus* panel and the potential value of cross-amplification strategies when finer-scale resolution is required. As a future direction, reassessing the genetic diversity and population structure of the species using high‑resolution genomic approaches, such as single nucleotide polymorphisms (SNPs), would likely yield more detailed insights into fine‑scale dispersal, gene flow, and introduction routes. Compared with SNPs, the relatively low number of microsatellite markers employed here represents a limitation that may have reduced the power to detect subtle population structure.

In contrast to microsatellites, the three analysed gene regions—nuclear ITS2 and the mitochondrial COI and ND4 fragments—revealed little genetic structure or geographic consistency among haplotypes across the Spanish range, with many haplotypes shared among distant countries, indicating that the available data are insufficient to resolve the origin of the Spanish *Ae. japonicus*. Such patterns, commonly observed in highly invasive species such as mosquitoes, suggest multiple human-mediated introductions from distinct source populations (e.g^46^.. for *Ae. albopictus*) and are not consistent with the microsatellite data. Nonetheless, it should also be noted that the mitochondrial and nuclear genes here analysed are known to evolve relatively slowly compared to microsatellites, providing limited resolution for detecting recent processes such as contemporary dispersal and invasion routes. Indeed, these markers are more informative for reconstructing ancient demographic events^72^. In contrast, the higher mutation rate of microsatellites generates higher allelic variability, offering greater power to detect recent demographic changes, fine-scale genetic structure, and short-term dispersal events. As a result, microsatellites outperform conventional gene fragments in uncovering patterns that might otherwise remain undetected.

Our screening of 112 *Ae. japonicus* specimens revealed no evidence of *Wolbachia* infection. *Wolbachia* is a maternally inherited endosymbiotic bacterium known to induce, among other effects, selective sweeps in mtDNA, i.e. the fixation of one or few haplotypes that may become widespread in the host population via cytoplasmic hitchhiking^29^. Selective sweeps have been shown to reduce mtDNA haplotype diversity, and may also cause departures from neutrality in the remaining set of haplotypes^73^. Within Culicidae, natural *Wolbachia* has been reported in more than 30 species, including several *Aedes* mosquitoes^45,46,74^. As for *Ae. japonicus*, recent findings suggest *Wolbachia* is rare^75,76^. Assessing *Wolbachia* presence was therefore necessary to rule out infection-driven selective sweeps as a confounding factor. The absence of *Wolbachia* in our dataset thus supports the reliability of the observed mtDNA patterns.

In conclusion, this study provides the most extensive population genetic assessment of *Ae. japonicus* conducted to date. The close genetic relatedness of most Spanish samples to geographically distant samples from the Baltimore area in the USA suggests a primary introduction route linking these populations, together with additional, smaller introduction pathways within Europe. Our results also reveal the presence of two main lineages within Europe, one centred in central-eastern Europe and another in central-western Europe. In addition to terrestrial dispersal mechanisms, which remain poorly understood and have only occasionally been directly linked to the commercial trade of used tires, our findings highlight the role of international seaports in facilitating mosquito invasions globally. These results underscore the need for continuous surveillance at key maritime points of entry to limit future introductions and mitigate the species’ ecological and public health impacts.

## Supplementary Information

Below is the link to the electronic supplementary material.

Supplementary Material 1

## Data Availability

Newly generated sequence data are available in GenBank (https://www.ncbi.nlm.nih.gov/genbank/) under the following accession numbers: PX924841- PX924843 for ITS2, PX916852-PX916866 for COI, and PX926782-PX926795 for ND4. Original sequence alignments and microsatellite genotypes are available in the Zenodo repository (https://doi.org/10.5281/zenodo.18313387).
