# Supplementary material for: Invasion dynamics of the disease vector Aedes japonicus in Spain
Source: Sci Rep. 2026 May 6;16:20733. doi: 10.1038/s41598-026-49121-x (PMC13333955; doi:10.1038/s41598-026-49121-x)
Supplement: Supplementary file 1 — Supplementary Material 1 [file 41598_2026_49121_MOESM1_ESM.pdf]

**Supplementary Information for:**

# Invasion dynamics of the disease vector *Aedes japonicus* in Spain

Federica Lucati, Fatima Chaoui, Maria Miranda Gómez, Jenny Caner, Katja Adam, Nikoleta Anicic, Karin Bakran-Lebl, Jesús F. Barandika, Manuel Barrón, Luisa Barzon, Norbert Becker, Aitor Cevidanes, Isra Deblauwe, Sarah Delacour-Estrella, Eleonora Flacio, Federica Gobbo, Mikel Alexander González, Adolfo Ibáñez-Justicia, Mihaela Kavran, Ana Klobučar, Marion Koopmans, Kornélia Kurucz, Paul T. Leisnham, Motoyoshi Mogi, Fabrizio Montarsi, Ignacio Ruiz-Arrondo, Francis Schaffner, Anna Schneider, Zoltán Soltész, Nobuko Tuno, Wim Van Bortel, Katie M. Westby, Roger Eritja, John Palmer, Frederic Bartumeus, Marc Ventura

## Tables

**Supplementary Table S1.** Geographic information of *Aedes japonicus* sampling sites, including collection date, sampling method, and life stage. 'NJ label' indicates labels used in the neighbour-joining tree (Fig. 5); 'Lat.' and 'Long.' indicate latitude and longitude in WGS84 coordinates. '$N_{\mu sat}$', '$N_{ITS2}$', '$N_{COI}$', and '$N_{ND4}$' indicate the number of samples analysed for microsatellites, ITS2, COI and ND4, respectively.

| Code | Country | Location | NJ label | Life stage | Collection date(s) | Lat. | Long. | Collection method | $N_{\mu sat}$ | $N_{ITS2}$ | $N_{COI}$ | $N_{ND4}$ |
|---|---|---|---|---|---|---|---|---|---|---|---|---|
| 1 | Austria | Althofen | Austria-1 | Larvae | 23-28/07/2021 | 46.87 | 14.47 | Larval dipping | 30 | 2 | 5 | 5 |
| 2 | | Weer | Austria-2 | Adults | 16-23/08/2023 | 47.31 | 11.65 | Egg sampling + rearing | 6 | | | |
| 3 | | Güssing | Austria-3 | Adults | 14-28/08/2023 | 47.08 | 16.34 | Egg sampling + rearing | 5 | | | |
| 4 | | Graz | Austria-3 | Adults | 16-23/08/2023 | 47.08 | 15.45 | Egg sampling + rearing | 6 | | | |
| 5 | | Deutschfeistritz | Austria-3 | Adults | 08-25/08/2023 | 47.20 | 15.32 | Egg sampling + rearing | 7 | | | |
| 6 | | Linz | Austria-4 | Adults | 14-23/08/2023 | 48.27 | 14.28 | Egg sampling + rearing | 4 | | | |
| 7 | Belgium | Maasmechelen | Belgium-1 | Adults | 17/07/2018 | 51.00 | 5.62 | Gravid traps | 14 | | | |
| 8 | | Natoye | Belgium-2 | Adults | 05/06/2023 | 50.34 | 5.05 | Larval dipping + rearing | 13 | | | |
| 9 | Croatia | Đurmanec | Croatia-1 | Larvae | 02/09/2022 | 46.20 | 15.84 | Larval dipping | 10 | | | |
| 10 | | Markuševec, Zagreb | Croatia-2 | Larvae | 06/08/2021 | 45.87 | 16.01 | Larval dipping | 4 | | | |
| 11 | | Planina gornja, Zagreb | Croatia-2 | Larvae | 21/09/2022 | 45.94 | 16.08 | Larval dipping | 1 | | | |
| 12 | | Donji Dragonožec, Zagreb | Croatia-2 | Larvae | 25/07/2022 | 45.66 | 15.95 | Larval dipping | 6 | | | |
| 13 | | Duga Resa | Croatia-3 | Larvae | 22/07/2021 | 45.45 | 15.50 | Larval dipping | 10 | | | |
| 14 | | Bjelovar | Croatia-4 | Larvae | 23/09/2021 | 45.75 | 16.62 | Larval dipping | 5 | | | |
| 15 | France | Steinbach | France | Larvae | 30/09/2021 | 47.82 | 7.15 | Larval dipping | 20 | 1 | 4 | 6 |
| 16 | Germany | Heidelberg | Germany | Larvae | 08/2021 | 49.41 | 8.69 | Larval dipping | 14 | | | |
| 17 | Hungary | Szalafő | Hungary-1 | Larvae | 02/09/2021 | 46.86 | 16.37 | Larval dipping | 9 | 1 | 3 | 1 |
| 18 | | Őriszentpéter | Hungary-1 | Larvae | 02/09/2021 | 46.85 | 16.40 | Larval dipping | 22 | 1 | 4 | 6 |
| 19 | | Kovácsszénája | Hungary-2 | Adults | 27/06/2021 - 25/08/2021 | 46.17 | 18.11 | Egg sampling + rearing | 20 | 2 | 1 | 3 |

| | | | | | | | | | | | | |
|---|---|---|---|---|---|---|---|---|---|---|---|---|
| 20 | Italy | Attimis | Italy | Adults | 10/06/2022 | 46.20 | 13.23 | Larval dipping + rearing | 19 | 3 | 4 | 4 |
| 21 | | Clauzetto | Italy | Larvae | 08/06/2022 | 46.24 | 12.91 | Larval dipping | 25 | 2 | 2 | 1 |
| 22 | | Travesio | Italy | Larvae | 08/06/2022 | 46.19 | 12.87 | Larval dipping | 4 | 4 | 4 | 4 |
| 23 | Japan | Saga | Japan-1 | Adults | 19/05/2018 | 33.33 | 130.27 | Larval dipping + rearing | 20 | 2 | | 3 |
| 24 | | Ishikawa | Japan-2 | Adults | 09/01/2021 | 36.55 | 136.70 | Larval dipping + rearing | 27 | 1 | 6 | 7 |
| 25 | | Sapporo | Japan-3 | Adults | 09/01/2021 | 43.07 | 141.34 | Larval dipping + rearing | 19 | 2 | 3 | 5 |
| 26 | Serbia | Sremska Kamenica | Serbia | Adults | 06/03/2023 | 45.19 | 19.85 | Egg sampling + rearing | 19 | | | |
| 27 | Slovenia | Bovec | Slovenia-1 | Larvae | 28/06/2022 | 46.34 | 13.55 | Larval dipping | 5 | | | |
| 28 | | Velike Žablje | Slovenia-1 | Larvae | 24/08/2021 | 45.87 | 13.85 | Larval dipping | 5 | | | |
| 29 | | Verd | Slovenia-2 | Larvae | 04/07/2022 | 45.97 | 14.31 | Larval dipping | 5 | | | |
| 30 | | Ilirska Bistrica | Slovenia-2 | Larvae | 04/07/2022 | 45.57 | 14.24 | Larval dipping | 5 | | | |
| 31 | | Olmo | Slovenia-2 | Larvae | 30/06/2022 | 45.53 | 13.72 | Larval dipping | 5 | | | |
| 32 | | Kočevje | Slovenia-3 | Larvae | 12/07/2022 | 45.64 | 14.85 | Larval dipping | 5 | | | |
| 33 | | Bizeljsko | Slovenia-3 | Larvae | 25/05/2020 | 46.02 | 15.70 | Larval dipping | 2 | | | |
| 34 | | Novo mesto | Slovenia-3 | Larvae | 12/06/2020 | 45.80 | 15.16 | Larval dipping | 5 | | | |
| 35 | Spain | Siero | Asturias | Adults + larvae | 20/07/2018 | 43.42 | -5.70 | Larval dipping + rearing | 27 | 1 | 5 | 6 |
| 36 | | Gijón | Asturias | Adults | 28/09/2019 | 43.54 | -5.61 | Larval dipping + rearing | 1 | | 1 | 1 |
| 37 | | Avín, Onís | Asturias | Adults | 27/09/2019 | 43.33 | -4.95 | Larval dipping + rearing | 1 | | | 1 |
| 38 | | Cardoso, Llanes | Asturias | Adults | 27/09/2019 | 43.44 | -4.92 | Larval dipping + rearing | 1 | | 1 | 1 |
| 39 | | Colombres, Pimiango | Asturias | Adults | 26/06/2019 | 43.38 | -4.55 | Larval dipping + rearing | 9 | | 1 | 1 |
| 40 | | Lieres, Siero | Asturias | Adults | 28/09/2019 | 43.39 | -5.58 | Larval dipping + rearing | 1 | | 1 | 1 |
| 41 | | Puenteles | Asturias | Adults | 26/09/2019 | 43.39 | -4.64 | Larval dipping + rearing | 13 | | 1 | 1 |
| 42 | | Robriguero | Asturias | Adults | 26/09/2019 | 43.35 | -4.60 | Larval dipping + rearing | 6 | | 1 | 1 |
| 43 | | Villanueva de Oscos | Asturias | Adults | 30/09/2019 | 43.52 | -6.42 | Larval dipping + rearing | | | | 1 |
| 44 | | Álava | Basque Country | Adults | 07-08/2020 | 42.66 | -2.51 | Egg sampling + rearing | 22 | 2 | 2 | 5 |

| | | | | | | | | | | | | |
|---|---|---|---|---|---|---|---|---|---|---|---|---|
| 45 | | Orio | Basque Country | Adults | 03/08/2021 | 43.29 | -2.11 | Larval dipping + rearing | 20 | 2 | 1 | 2 |
| 46 | | Zamudio | Basque Country | Adults | 03/05/2021 | 43.30 | -2.87 | Larval dipping + rearing | 20 | | 1 | 6 |
| 47 | | Altzola, Elgoibar | Basque Country | Adults | 07/06/2020 | 43.24 | -2.40 | Larval dipping + rearing | 19 | | 1 | 2 |
| 48 | | Santa Klara, Elgoibar | Basque Country | Adults | 14/06/2020 | 43.22 | -2.42 | Larval dipping + rearing | 5 | | 3 | 3 |
| 49 | | Polígono industrial, Elgoibar | Basque Country | Adults | 07/05/2020 | 43.21 | -2.41 | Larval dipping + rearing | 2 | | 1 | 1 |
| 50 | | Alto de Azcarate, Elgoibar | Basque Country | Adults | 06/04/2020 | 43.20 | -2.36 | Larval dipping + rearing | 1 | | 1 | 1 |
| 51 | | Goiuria, Yurreta | Basque Country | Adults | 07/05/2020 | 43.19 | -2.64 | Larval dipping + rearing | 3 | | 1 | 1 |
| 52 | | Saja, Los Tojos | Cantabria | Adults | 26/09/2019 | 43.35 | -4.10 | Larval dipping + rearing | 2 | | 1 | 1 |
| 53 | | Ruente | Cantabria | Adults | 26/09/2019 | 43.26 | -4.27 | Larval dipping + rearing | 1 | | 1 | 1 |
| 54 | | Bera de Bidasoa | Navarre | Adults | 03/07/2023 | 43.27 | -1.70 | Larval dipping + rearing | 6 | | | |
| 55 | | Bertiz | Navarre | Adults | 03/07/2023 | 43.14 | -1.61 | Larval dipping + rearing | 6 | | | |
| 56 | Switzerland | Morcote | Switzerland | Adults | 05/01/2023 | 45.93 | 8.90 | Egg sampling + rearing | 19 | | | |
| 57 | The Netherlands | Lelystad | The Netherlands-1 | Adults and larvae | 24/06/2021 - 14/03/2022 | 52.53 | 5.47 | Larval dipping + rearing | 27 | 1 | 5 | 5 |
| 58 | | Urk | The Netherlands-1 | Larvae | 14/03/2022 | 52.67 | 5.61 | Larval dipping | 5 | | | |
| 59 | | Vlodrop | The Netherlands-2 | Larvae | 16/03/2022 | 51.15 | 6.15 | Larval dipping | 10 | | | |
| 60 | | Roermond | The Netherlands-2 | Larvae | 16/03/2022 | 51.19 | 6.06 | Larval dipping | 5 | | | |
| 61 | USA | Saint Louis, MO | USA-1 | Adults | 16/08/2021 | 38.53 | -90.56 | Egg sampling + rearing | 19 | | 3 | 4 |
| 62 | | College Park, MD | USA-2 | Adults | 09/10/2021 | 38.99 | -76.94 | Egg sampling + rearing | 8 | 4 | 2 | 5 |

**Supplementary Table S2.** *Aedes japonicus*/*albopictus* microsatellite set employed in the present study. For each microsatellite, the repeat motif, allele size range, forward (F) and reverse (R) primer sequences, primer concentration ($C_0$), amplification multiplex panel (MPX) and primer label are shown. [1] *Ae. japonicus* microsatellites from [1]; [2] *Ae. albopictus* microsatellites from [2]; [3]*Ae. albopictus* microsatellites from [3]. Loci Alb-tri-41 and Aealbmic13 were discarded from analyses due to poor amplification.

| Locus | Repeat motif | Allele size range (bp) | Primer sequences | $C_0$ ($\mu$M) | MPX | Primer label (fluorescent dye) |
|---|---|---|---|---|---|---|
| OJ10[1] | (GTT)(GTG)(GTT)8 | 110-139 | F: GCTTGTCCTGGCTAAGTACTGC<br>R: CGGTAATGTCCACCTGATTG | 0.2 | 1 | 6-FAM |
| OJ70[1] | (GCT)4(GTT)2(GCT)2(GTT)<br>(GCT)2(GTT)2(GCT)7 | 184-215 | F: CGTTGACAAAGCTCATCTGC<br>R: TGATCTCCAACGGAAGTATGC | 0.2 | 1 | 6-FAM |
| OJ5[1] | (GTT)6(GCT)3(GTT) | 141-157 | F: CACGAAGTCTGGAAGATCTGG<br>R: ATTCGTGCAGCGAAATCTG | 0.2 | 1 | 6-FAM |
| OJ187[1] | (CGA)11 | 120-164 | F: AAATCAGCTGCCAGTGCAAG<br>R: TGTGTACTTTGCGGTGAAGG | 0.2 | 1 | VIC |
| OJ100[1] | (GT)5 | 174-202 | F: CGCATTCCTCAAACCCTAAC<br>R: TCGGTCCGAGGGAAAAAC | 0.2 | 1 | VIC |
| OJ85[1] | (CAG)6 | 161-182 | F: CATAAAGCAGCAAGCACAGC<br>R: TGTCTTCCGGATTGATTTCC | 0.2 | 1 | NED |
| OJ338[1] | (CAA)10 | 134-185 | F: TCTCCTGATCCTGAAGAAGC<br>R: AGGGAGCAGAGCAACACTTG | 0.2 | 1 | PET |
| Alb-tri-6[2] | (AGC)n | 143-188 | F: AGCACGAGTACAGAATGTGC<br>R: TGGCCTCCTACCGTTTATCTG | 0.3 | 2 | 6-FAM |
| Aealbmic11[3] | (TGT)n | 188-230 | F: CTCTGCGTTCCGGTTCTATC<br>R: AGGCAACCTCTCGAATGAAA | 0.3 | 2 | 6-FAM |
| Alb-tri-33[2] | (GGC)n | 118-144 | F: GGCTGCTGTTGTTGGTACG<br>R: CACGTTCAATCACCGGTTCC | 0.3 | 2 | VIC |
| Alb-tri-46[2] | [TTC]n | 145-182 | F: TTCACAACATACGGAATCGC<br>R: GGTCCGGTGTAATAGCCTCC | 0.3 | 2 | VIC |
| Alb-tri-44[2] | (CAC)n | 161-197 | F: CACTCGCGCGTGTTCTTC<br>R: GACGCACCATCAGCATCATC | 0.3 | 2 | NED |
| Alb-tri-25[2] | (CCAA)n | 247-260 | F: CCAACCAACAACCCAGGAAC<br>R: TACGATGCGCAACCATCATC | 0.3 | 2 | NED |
| Alb-tri-41[2] | (GAT)n | 100-160 | F: GATCGATTTGGGAGCTTCTG<br>R: GAACCTCTTCTCGCTTGGCT | 0.3 | 2 | PET |
| Aealbmic5[3] | (TGT)n | 136-220 | F: AACCCATCGAACACAGAAGG<br>R: GTACGGTTGACTCGCTGTGA | 0.3 | 3 | 6-FAM |
| Aealbmic13[3] | (GAT)GAC(GAT)n | 123-171 | F: TCACACCATGGTCAAAGCAT<br>R: TGCTGAGTTGAATGGAAACG | 0.3 | 3 | VIC |
| Alb-tri-45[2] | (TTT)n | 110-136 | F: TTTCAGCTCGGTGTTATGGC<br>R: TGATGTTGATGATGATGACTACGA | 0.3 | 3 | NED |
| Alb-tri-20[2] | (GTG)n | 145-184 | F: GTGCCGTTGATCATCCTGTC<br>R: TCCAGCACCGTGAGTAATCC | 0.3 | 3 | NED |
| Aealbmic3[3] | (AAC)n | 200-239 | F: ACCATACAGCCTGGAGTTCG<br>R: GGGGTTGTGTGAATTGTCGT | 0.3 | 3 | NED |
| Aealbmic7[3] | (TTG)nATG(TTG)n | 188-220 | F: ATAGACGGGAGTCGGTTCCT<br>R: TCCAACCGCTAGTGTCATCA | 0.3 | 3 | PET |

## Figures

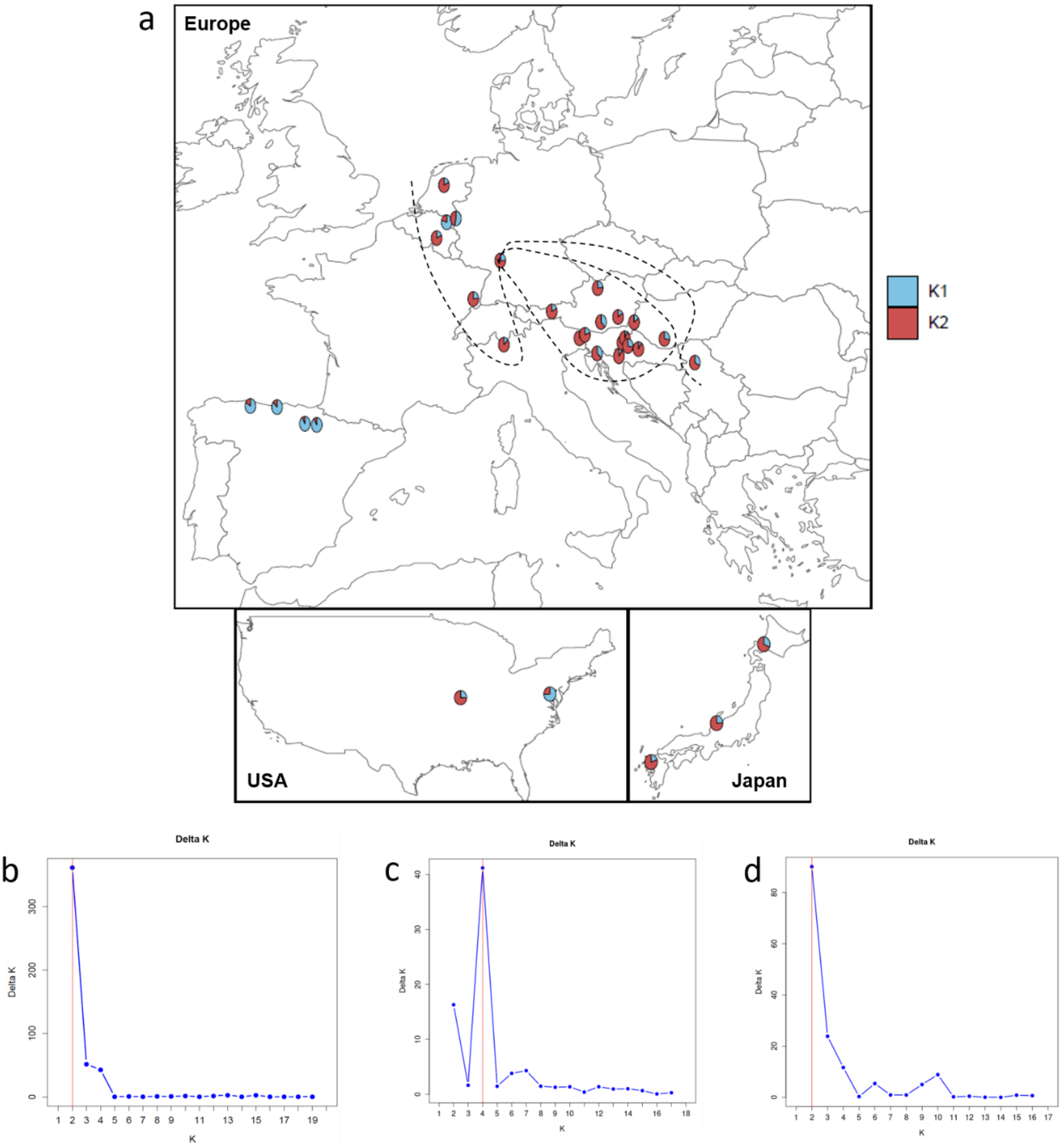


**Supplementary Figure S1.** Results of Bayesian clustering analysis (STRUCTURE). (a) Results of STRUCTURE for K = 2 microsatellite groups for *Aedes japonicus*. Sampled populations are represented by pie charts highlighting the population cluster membership obtained in STRUCTURE. Samples were grouped at the regional level to facilitate visualization (see Table S1). Dashed lines separate the two sub-clusters found within the main red group. (b), (c) and (d) $\Delta K$ values as a function of K, calculated according to [4], for the total number of sampled sites and for the first (light blue) and second (red) genetic clusters, respectively.

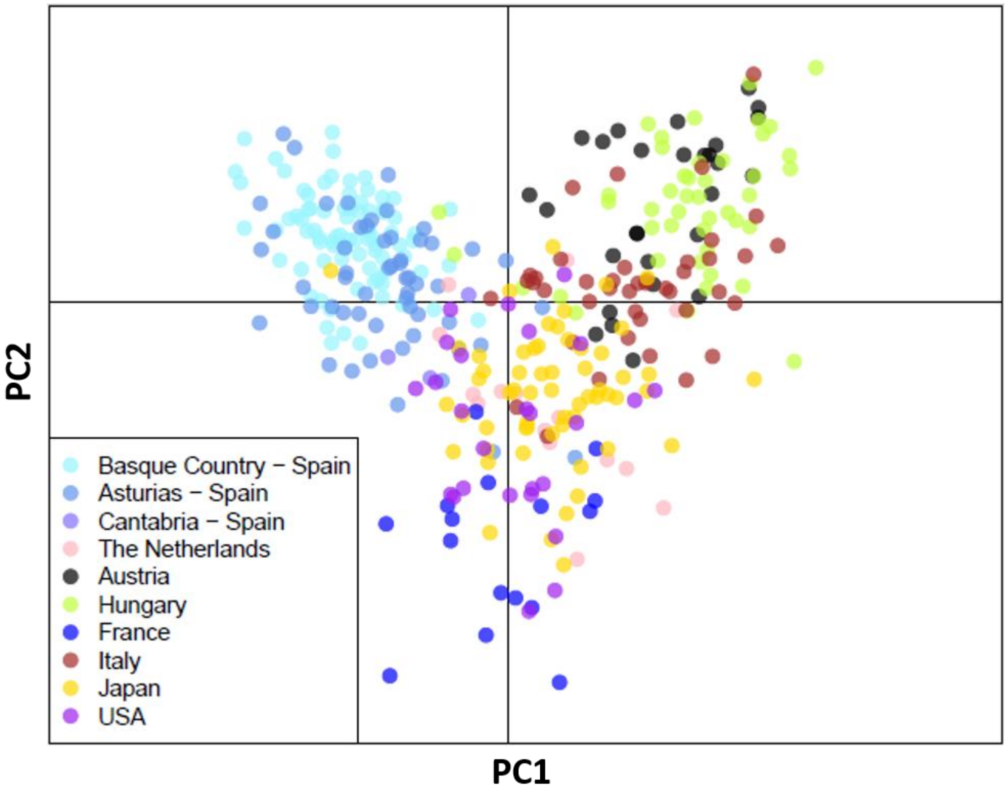


**Supplementary Figure S2.** Principal Component Analysis (PCA) of a subset of *Aedes japonicus* samples, based on the combination of *Ae. japonicus* and *Ae. albopictus*-specific microsatellites as shown in Table S2. Each point represents a single individual. Samples are coloured according to their geographic origin—by region for Spanish populations and by country for all others.

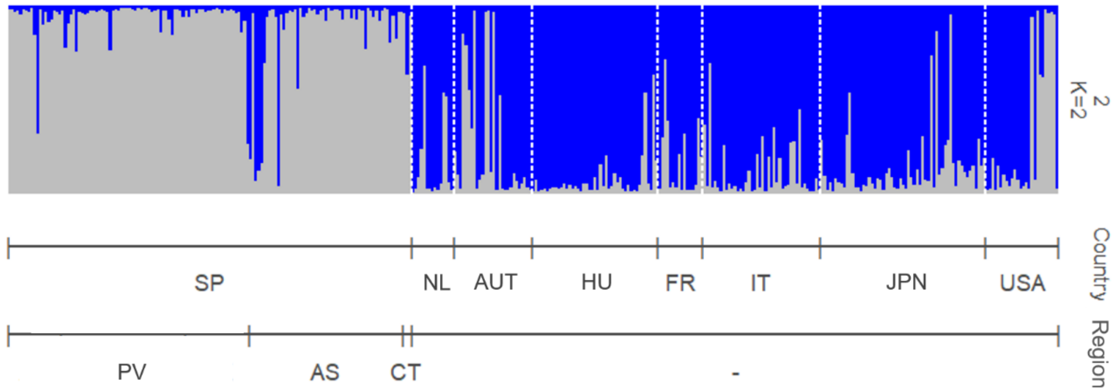


**Supplementary Figure S3.** STRUCTURE barplot showing individual membership probabilities for K = 2 in a subset of *Aedes japonicus* samples, based on the combination of *Ae. japonicus* and *Ae. albopictus*-specific microsatellites (see Table S2). Each individual is represented by a vertical bar corresponding to the sum of assignment probabilities to the K cluster. White dashed lines separate countries. Country/Spanish region codes: SP: Spain; NL: The Netherlands; AUT: Austria; HU: Hungary; FR: France; IT: Italy; JPN: Japan; USA: United States; PV: Basque Country; AS: Asturias; CT: Cantabria.

**References**


1 Widdel, A. K., McCuiston, L. J., Crans, W. J., Kramer, L. D. & Fonseca, D. M. Finding needles in the haystack: single copy microsatellite loci for *Aedes japonicus* (Diptera: Culicidae). *Am. J. Trop. Med. Hyg.* **73**, 744 (2005).

2 Beebe, N. W. *et al.* Tracing the tiger: population genetics provides valuable insights into the *Aedes* (*Stegomyia*) *albopictus* invasion of the Australasian region. *PLoS Negl. Trop. Dis.* **7**, e2361 (2013).

3 Manni, M. *et al.* Molecular markers for analyses of intraspecific genetic diversity in the Asian tiger mosquito, *Aedes albopictus*. *Parasit. Vectors* **8**, 188 (2015).

4 Evanno, G., Regnaut, S. & Goudet, J. Detecting the number of clusters of individuals using the software STRUCTURE: A simulation study. *Mol. Ecol.* **14**, 2611-2620 (2005).
